# Redox Biology of Right-Sided Heart Failure

**DOI:** 10.3390/antiox7080106

**Published:** 2018-08-08

**Authors:** Nataliia V. Shults, Oleksiy Melnyk, Dante I. Suzuki, Yuichiro J. Suzuki

**Affiliations:** Department of Pharmacology and Physiology, Georgetown University Medical Center, 3900 Reservoir Road NW, Washington, DC 20007, USA; ns1015@georgetown.edu (N.V.S.); oleksiym@ymail.com (O.M.); dantealessiajustin@gmail.com (D.I.S.)

**Keywords:** antioxidants, redox, reactive oxygen species, right heart failure

## Abstract

Right-sided heart failure is the major cause of death among patients who suffer from various forms of pulmonary hypertension and congenital heart disease. The right ventricle (RV) and left ventricle (LV) originate from different progenitor cells and function against very different blood pressures. However, differences between the RV and LV formed after birth have not been well defined. Work from our laboratory and others has accumulated evidence that redox signaling, oxidative stress and antioxidant regulation are important components that define the RV/LV differences. The present article summarizes the progress in understanding the roles of redox biology in the RV chamber-specificity. Understanding the mechanisms of RV/LV differences should help develop selective therapeutic strategies to help patients who are susceptible to and suffering from right-sided heart failure. Modulations of redox biology may provide effective therapeutic avenues for these conditions.

## 1. Introduction

Patients with pulmonary hypertension and repaired congenital heart disease are at risk for developing right-sided heart failure [1,2,3]. However, the pathogenic mechanism of right heart failure is not well understood [4]. Much of the knowledge concerning the biology of the heart has been derived from the studies of the left ventricle (LV) and it has generally been assumed that the biology of the right ventricle (RV) is identical to that of the LV. However, some studies have revealed that mechanisms of right and left heart failure may be different. In the LV, concentric hypertrophy in response to systemic hypertension is often followed by the transition to dilation with eccentric cardiac hypertrophy and thinning of the ventricular wall. By contrast, in pulmonary hypertension, the concentrically hypertrophied RV appears to undergo failure, manifested by a well-known pathological observation of cor pulmonale [5,6]. Further, it is unclear whether therapies that were developed to treat LV failure really benefit patients with right-sided heart failure. Thus, understanding the differences between the RV and LV should contribute to the development of new therapeutic strategies.

Developmentally, LV and RV myocytes are derived from different precursor cells. Cells in the first heart field (primary heart field) contribute to the formation of the LV myocardium, whereas cells in the second heart field (anterior heart field) construct the RV myocardium [7,8,9]. Functionally, unlike the LV, the RV pumps the blood against a wide range of pressures throughout life (~100 mmHg in utero and ~10 mmHg after birth). However, the overall protein expression patterns of the adult RV and LV free walls were found to be remarkably similar [10]. Defining the subtle differences between the two ventricles may promote the development of therapeutic strategies that are tailored to specific pathologic conditions. Our laboratory previously identified some differences in protein expression between the RV and LV [11,12], as well as differentially regulated signal transduction pathways [12,13]. Notably, these studies revealed the importance of redox regulation in the RV to LV differences. This article summarizes our studies as well as ones by others, which describe redox biology of right-sided heart failure. 

## 2. Oxidative Modifications in Right-Sided Heart Failure

In rats, the injection of an inhibitor of the vascular endothelial growth factor receptor (SU5416) plus stimuli such as hypoxia and ovalbumin immunization trigger pulmonary arterial hypertension [14,15] and right heart failure [16,17]. It was found that failing RVs in response to SU5416/ovalbumin-induced pulmonary hypertension are subjected to specific types of protein oxidation. Total protein *S*-glutathionylation, nitrotyrosine formation, and *S*-nitrosocysteine formation were found to be higher in the failing RV compared to control RV [17]. Mass spectrometry identified that these *S*-nitrotyrosinylated proteins include heat shock protein-90 and sarcoplasmic reticulum Ca^2+^-ATPase, and *S*-glutathionylated proteins include heat shock protein-90 and NADH-ubiquinone oxidoreductase [17].

Total protein carbonylation was not altered in the RVs of rats with pulmonary hypertension compared with the controls [17]. However, our metabolomics analysis revealed that peptides that contain susceptible amino acids for carbonylation, that is, arginine, lysine, proline and threonine (as previously described by Berlett and Stadtman [18]), were lower in the RVs of pulmonary hypertensive rats than in controls [19]. Twenty-eight peptides were identified to be significantly decreased at least two-fold in the RVs of pulmonary hypertensive rats (Table 1). Notably, the Phe-Lys-Lys peptide was found to be more than 40-fold lower in the RVs of rats with pulmonary hypertension than in the RVs of healthy rats. Among these 28 peptides, 24 contained at least one carbonylation-susceptible amino acid. Among 112 amino acids within these 28 peptides, 51 amino acids (over 45%) were identified as carbonylation-susceptible amino acids that comprise four amino acids out of 20 (that is, 20%). 

## 3. RV-Specific Redox Regulation of GATA4 Gene Expression

**Figure 1:**
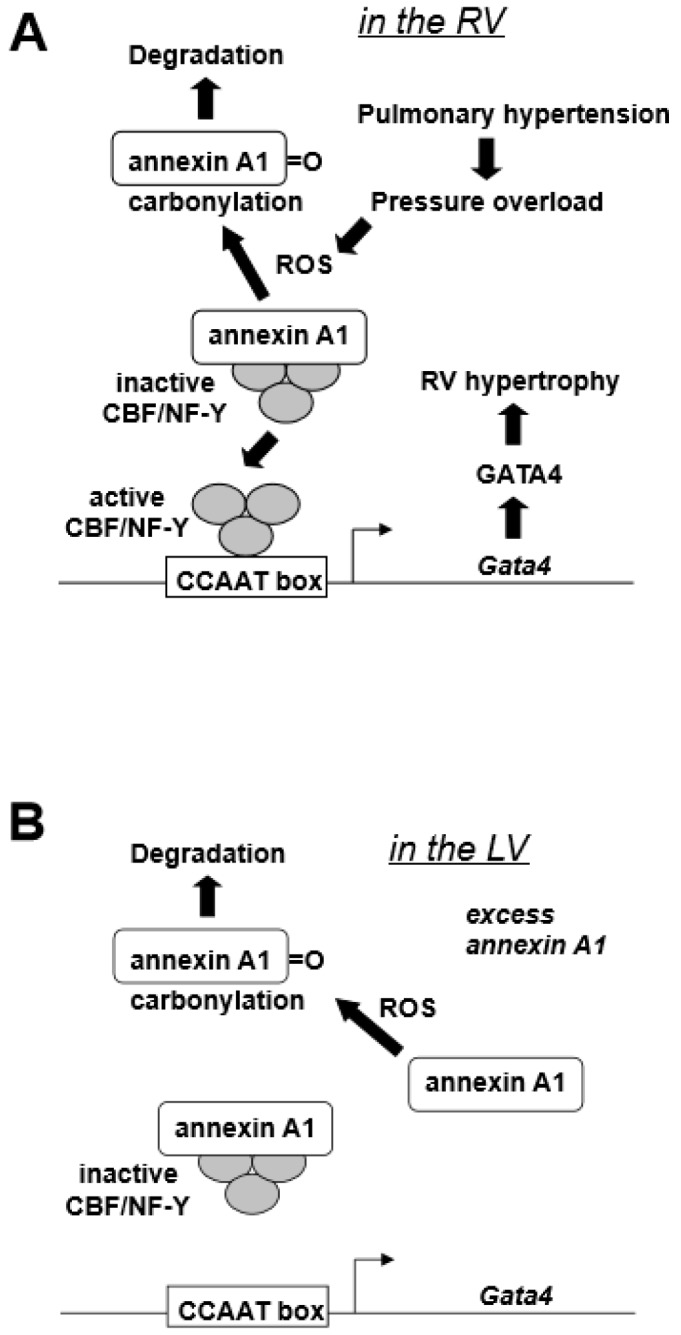
Proposed right ventricles (RV)-specific GATA4 activation mechanism. (**A**) Pulmonary hypertension produces reactive oxygen species (ROS) in the RV that in turn promote the carbonylation of annexin A1 protein. Carbonylation elicits the annexin A1 degradation by proteasomes, liberating CBF/NF-Y transcription factor that activates *Gata4* gene transcription [12]. (**B**) In the left ventricle (LV), the low CBF/NF-Y-to-annexin A1 ratio with excess annexin A1 does not allow for the efficient CBF/NF-Y activation.

GATA4, a major transcription factor in the regulation of cardiac hypertrophy [20], is activated through post-translational modification mechanisms in the LV [21]. In our previously studies [12], the RV GATA4 DNA binding activity was found to be increased in a rat model of pulmonary hypertension. However, this was not because of the post-translational activation of this protein, but due to increased gene transcription, since both protein and mRNA levels of GATA4 were also increased in response to pulmonary hypertension in the RV, but not in the LV [12]. Our laboratory cloned the *Gata4* promoter [22] and found that CBF/NF-Y binding to CCAAT box mediates the increased *GATA4* gene expression [12]. Annexin A1 was found to interact with CBF/NF-Y during pulmonary hypertension-mediated RV hypertrophy and negatively regulate CBF/NF-Y DNA binding [12]. Further, annexin A1 gets degraded in the RV, but not in the LV, in response to pulmonary hypertension, indicating that the activation of CBF/NF-Y-dependent *GATA4* gene transcription is through releasing the negative regulation by annexin A1 [12]. This RV-specific mechanism of GATA4 activation that is dependent on the activation of gene transcription may be defined by the difference in the expression levels of CBF/NF-Y as the RV has more CBF-B compared to the LV, while the annexin A1 levels were comparable between the two ventricles [12]. The finding that annexin A1 is involved in this mechanism of RV hypertrophy was of great interest in terms of redox signaling biology because our laboratory previously found a mechanism that involves the proteasome-dependent degradation of annexin A1 in response to protein carbonylation in smooth muscle cells [23]. Annexin A1 was found to be also carbonylated in response to pulmonary hypertension in the RV, but not in the LV [12]. As an RV-specific mechanism of cell signaling, our laboratory proposed the “oxidation/degradation pathway of signal transduction” involving carbonylation and subsequent degradation of annexin A1 by proteasomes that regulate CBF/NF-Y-dependent *GATA4* gene expression (Figure 1A) [12]. Table 2 summarizes a series of experimental observations that led to this proposed mechanism. The higher CBF/NF-Y-to-annexin A1 ratio allows for the increased sensitivity for the CBF/NF-Y activation as annexin A1 gets degraded, hence conferring the RV-specificity of this mechanism. Figure 1B depicts that, under low CBF/NF-Y-to annexin-A1 ratio, ROS-dependent annexin A1 degradation does not allow for efficient CBF/NY-Y activation to promote *GATA4* gene expression.

It was also found that global cardiac ischemia/reperfusion injury results in reduced expression of GATA4 in the RV, but not in the LV [13]. Our laboratory previously established that GATA4 plays an important role in regulating the gene expression of an anti-apoptotic protein Bcl-x_L_ in cardiomyocytes [24,25]. Consistently, the RV-specific downregulation of GATA4 by ischemia/reperfusion seems to reflect Bcl-x_L_ gene expression, as global myocardial ischemia/reperfusion downregulated Bcl-x_L_ in the RV, but not in the LV [13].

## 4. The RV-Specific Redox Regulation of Serotonin Signaling

Our laboratory also discovered that serotonin promoted protein carbonylation in the RV, but not in the LV [11]. RV and LV homogenates derivatized with 2,4-dinitrophenylhydrazine (DNPH) exhibit multiple carbonylated proteins. Perfusing the isolated rat heart with serotonin for 10 min increased carbonylation of various proteins in the RV, but not in the LV [11]. It was concluded that the mechanism of this RV/LV difference is because monoamine oxidase-A is less expressed in the RV [11]. Thus, the intracellular serotonin degradation action of monoamine oxidase-A may trigger serotonin-induced protein carbonylation in the RV.

## 5. Metabolomics Analysis to Define the Difference between the RV and LV

**Figure 2:**
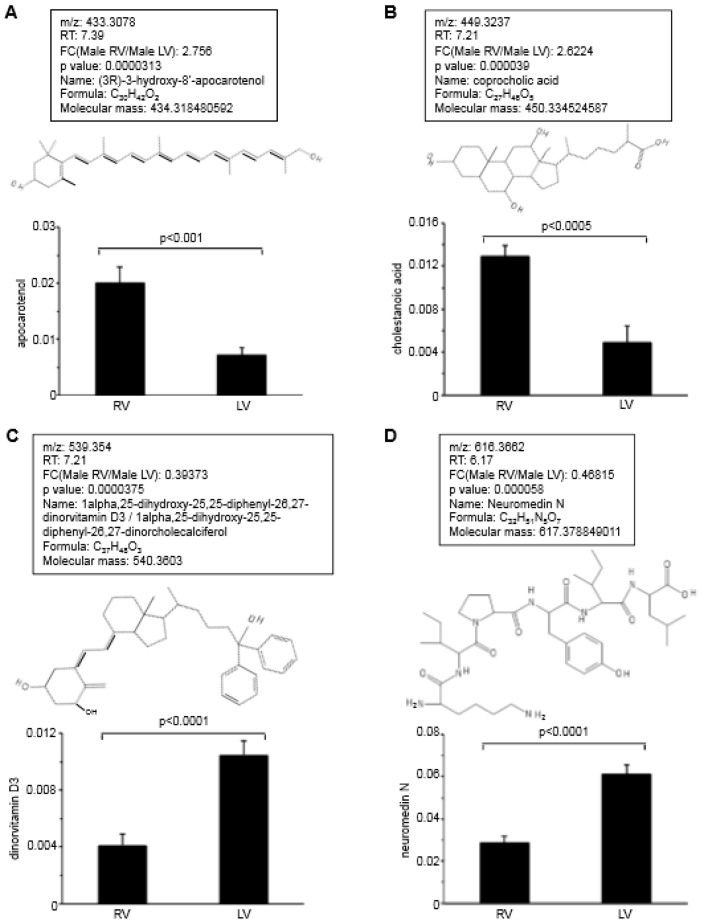
Notable metabolites differentially expressed between the RV and LV. The RV and LV wall tissues were dissected from male Sprague Dawley rats and subjected to metabolomics analysis (*n* = 10). The analysis of metabolomics data revealed 4 biologically notable molecules including (**A**) (3*R*)-3-hydroxy-8′-apocarotenol, (**B**) coprocholic acid, (**C**) 1alpha,25-dihydroxy-25,25-diphenyl-26,27-dinorvitamin D3 and (**D**) neuromedin N that exhibited significant differences in the RV and LV. For each molecule, the experimental *m*/*z* values (which represent mass), retention time (RT), fold-change (FC) ratio of RV to LV, *p*-values, name of the molecule, empirical formula, mass of the molecule and the chemical structures are noted. Bar graphs represent means ± SEM of the intensity values obtained from mass spectra.

To investigate the possible differences between the adult RV and LV, metabolomics analysis was performed using RV and LV free wall tissues obtained from adult rats by examining molecules with mass less than 1000. While a majority of metabolites seem to be expressed at similar levels between the RV and LV, some molecules exhibited statistically significant difference between their levels in the two ventricles. From these data, four biologically relevant molecules that occur at different levels between the RV and LV were identified. Among them, the levels of (3*R*)-3-hydroxy-8′-apocarotenol (Figure 2A) and coprocholic acid (Figure 2B) were found to be higher in the RV than in the LV. By contrast, 1alpha,25-dihydroxy-25,25-diphenyl-26,27-dinorvitamin D3 (Figure 2C) and neuromedin N (Figure 2D) were found to occur at lower levels in the RV compared to the LV. These identified molecules may exhibit redox properties. (3*R*)-3-hydroxy-8′-apocarotenol has potential to confer a similar activity as β-carotene. In addition, it possesses a hydroxyl group that may be redox active. Coprocholic acid and 1alpha,25-dihydroxy-25,25-diphenyl-26,27-dinorvitamin D3 also contain four and three hydroxyl groups, respectively.

Neuromedin N and another larger peptide, neuromedin N, both elicit cell signaling by activating neurotensin receptors. Neuromedin N is a small peptide with six amino acids, Lys-Ile-Pro-Tyr-Ile-Leu, while neurotensin contains 13 amino acids, Glu-Leu-Tyr-Glu-Asn-Lys-Pro-Arg-Arg-Pro-Tyr-Ile-Leu. Interestingly, both peptides are relatively rich in carbonylation-susceptible amino acids described above. 33% of amino acids in neuromedin N and 38% in neurotensin are composed of carbonylation-susceptible amino acids.

**Figure 3:**
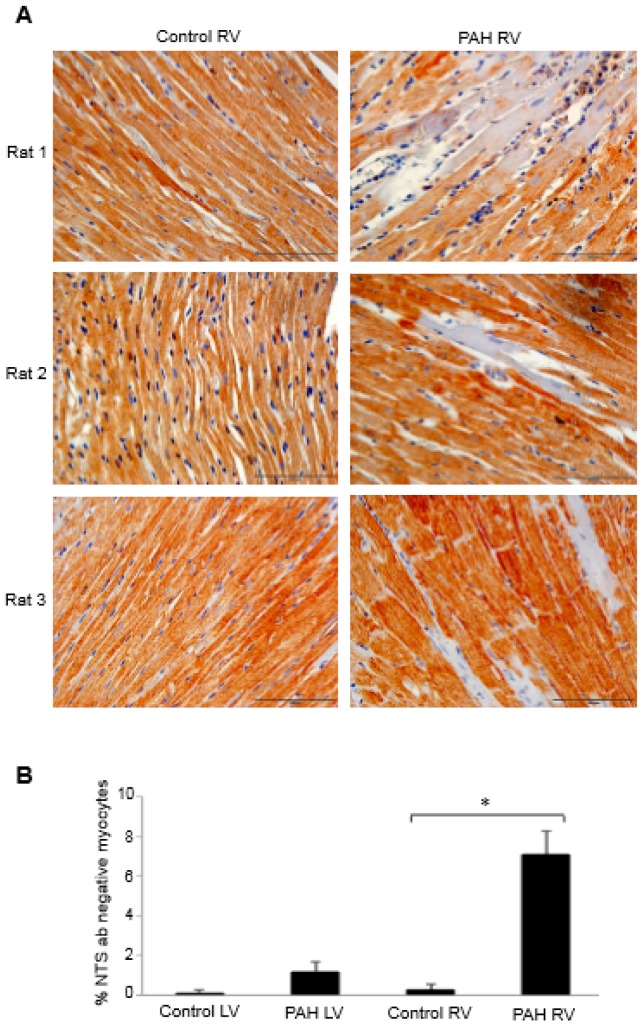
Neurotensin/neuromedin N expression in the heart of rats with pulmonary arterial hypertension (PAH). Male Sprague Dawley rats were subcutaneously injected with SU5416 (20 mg/kg body weight), maintained in hypoxia in a chamber set to maintain 10% O_2_ [12,26] for three weeks and then in normoxia for 17 weeks [27,28]. Heart tissues were immersed in buffered 10% formalin, embedded in paraffin, cut and mounted on glass slides. Tissue sections were subjected to immunohistochemistry using the neurotensin/neuromedin N (NTS) antibody (catalog # MBS8505326; MyBioSource, Inc., San Diego, CA, USA). (**A**) Representative immunohistochemistry images of the RVs from 3 pairs of control and PAH animals. (**B**) The bar graph represents means ± SEM of % NTS antibody-negative cells in the LV and RV. * denotes values significantly different from each other at *p* < 0.001.

To investigate the possible role of neurotensin/neuromedin N in right heart failure, immunohistochemistry experiments using the neurotensin/neuromedin N antibody were performed to compare the RVs of control rats to those of rats with right heart failure promoted by the injection of SU5416 plus chronic hypoxia that promotes severe pulmonary hypertension [26]. The examination of immunohistochemistry data revealed the occurrence of some RV myocytes that do not stain with the neurotensin/neuromedin N antibody. Figure 3A shows such observations from multiple animals. By contrast, the LV from pulmonary hypertension rats did not exhibit this phenomenon. Quantifications of the number of myocytes that did not stain with the neurotensin/neuromedin N antibody determined that the RVs of pulmonary hypertensive rats only exhibit such cells (Figure 3B). Thus, it appears that pressure overload to the RV in response to pulmonary hypertension modified some of cardiomyocytes so that they no longer express neurotensin or neuromedin N. In the context of redox biology, it would be interesting and potentially important to determine the carbonyl status of these peptides in the future.

## 6. RV/LV Differences in Oxidative Stress and Antioxidant Defense

Studies of experimental animals and human patients revealed that the RV is more susceptible to the occurrence of oxidative stress than the LV in the setting of heart failure because the RV has weaker antioxidant-adaptive defense mechanisms [3,29,30].

In female Wistar rats, Schreckenberg et al. [29] found that the RV is subjected to higher oxidative stress as detected by dihydroethidium staining for superoxide and measurements of peroxynitrite using an enzyme-linked immunosorbent assay. Differential superoxide levels between the two ventricles may be due to varied expression of manganese superoxide dismutase. In this study, using nitric oxide deficiency as a model of heart failure, authors concluded that the RV cannot cope with oxidative stress because this ventricle lacks the ability to upregulate manganese superoxide dismutase.

In human heart failure patients, Borchi et al. [30] reported that NADPH oxidase-dependent production of superoxide is higher in the RV compared to the LV. Lipid peroxidation as assessed by measuring the levels of malondialdehyde was also higher in the RV of failing hearts compared to the LV. Taken together with their data on antioxidant enzyme activities, authors concluded that oxidative stress promotes antioxidant-adaptive responses more in the LV compared to the RV.

In children with tetralogy of Fallot, Chaturvedi et al. [31] observed RV oxidative stress after the surgical repair. These authors concluded that patients destined to develop acute RV dysfunction and log intensive care unit stays after tetralogy of Fallot repair suffer more oxidative stress. Reddy et al. [32] further reported that, in children with tetralogy of Fallot, the RV fails to adapt to hypoxic stress. In their study, the expression of glutathione peroxidase was found to be lower in the RV of patients with lower O_2_ saturations.

## 7. Conclusions

The RV health is crucial to patients with pulmonary hypertension as well as with congenital heart disease including tetralogy of Fallot, pulmonary atresia, transposition of the great arteries and hypoplastic left heart syndrome. While the global RV and LV gene expression patterns and cell signaling mechanisms are similar, there are some subtle but crucial differences between RV and LV that may potentially define chamber-specific therapeutic strategies. Redox signaling, oxidative stress status and antioxidant regulation define important RV/LV differences. Further work focusing on these biological mechanisms may improve preventative and/or treatment strategies to help patients who are susceptible to developing right-sided heart failure. We hope that this article will promote further basic research concerning redox mechanisms of RV failure and possibly future clinical trials for the use of redox agents for the treatment of right-sided heart failure.

## Figures and Tables

**Table 1:** Metabolomics studies identified peptides that are lower in right ventricles (RVs) of Sprague Dawley rats with pulmonary arterial hypertension (PAH) than in controls. *p* < 0.05 (*n* = 10). Most of the peptides contain carbonylation-susceptible amino acids (AAs) indicated in bold.

Peptides	Fold Difference (Control/PAH)	Total # of AAs	Carbonylation Susceptible AAs	
			#	%
Glu Ile **Lys Pro**	4.8	4	2	50
Asp **Lys Lys Pro**	2.5	4	3	75
**Lys Arg Thr Thr**	2.2	4	4	100
Phe Gly **Arg Arg**	4.5	4	2	50
Ser Val **Lys Arg**	2.5	4	2	50
**Lys** Trp **Lys**	2.0	3	2	67
**Lys** Tyr Ile Glu	2.7	4	1	25
Ser Leu Leu Ser Phe	2.2	5	0	0
Asp Leu Phe **Arg**	2.4	4	1	25
**Thr Thr** Gly Leu Ile	2.8	5	2	40
**Lys** Tyr **Thr Arg**	2.5	4	3	75
**Arg** Ser **Lys Arg**	3.0	4	3	75
Trp Phe Trp	2.3	3	0	0
Asn **Arg** Phe **Lys**	2.8	4	2	50
His Ile Ile Val	3.1	4	0	0
**Arg Lys Lys** Cys	3.0	4	3	75
Asn **Arg** Phe **Lys**	3.2	4	2	50
Phe Ile Gln **Lys**	3.0	4	1	25
Ala **Arg** Tyr **Arg**	2.6	4	2	50
Ala Ala Ile **Lys**	4.8	4	1	25
Glu Phe **Pro** Trp	2.3	4	1	25
Phe **Thr Thr Thr**	2.2	4	3	75
Val **Arg** His **Arg**	2.6	4	2	50
Ile Ile Val Tyr	2.2	4	0	0
**Pro** Gln **Arg Thr**	3.0	4	3	75
Phe **Lys Lys**	41.7	4	2	50
**Thr Thr** Gly Leu Ile	2.5	4	2	50
Glu **Lys** Ala **Arg**	2.1	4	2	50

#: number.

**Table 2:** List of experimental observations for the RV-specific mechanism of GATA4 activation regulated by redox signaling.

Pulmonary hypertension activates GATA4 DNA binding activity in the RV.
Pulmonary hypertension increases GATA4 protein expression in the RV.
Pulmonary hypertension increases *Gata4* mRNA expression in the RV.
The *Gata4* promoter contains a functionally important CCAAT box.
The CBF/NF-Y transcription factor regulates CCAAT box of the *Gata4* promoter.
CBF/NF-Y binds to annexin A1.
Pulmonary hypertension promotes the degradation of annexin A1.
The degradation of annexin A1 is regulated by metal-catalyzed oxidation of annexin A1.
The RV has higher CBF/NF-Y-to-annexin A1 ratio than the left ventricle (LV)
